# Validation of the Geriatric Oral Health Assessment Index in Finland: A Questionnaire Study

**DOI:** 10.1002/cre2.70334

**Published:** 2026-03-26

**Authors:** Olli Koskela, Annamari Nihtilä, Marja Äijö, Kaarina Sirviö, Tiina Lampi, Anna Liisa Suominen

**Affiliations:** ^1^ Institute of Dentistry University of Eastern Finland Kuopio Finland; ^2^ Finnish Dental Society Apollonia Helsinki Finland; ^3^ School of Health Care Savonia University of Applied Sciences Kuopio Finland; ^4^ Wellbeing Services County of North Savo Kuopio Finland; ^5^ Oral and Maxillofacial Teaching Unit, Primary Oral Health Care Services, Wellbeing Services County of North‐Savo Kuopio Finland

**Keywords:** Geriatric Oral Health Assessment Index (GOHAI), older adults, oral health‐related quality of life, validation

## Abstract

**Objectives:**

The aim of this study was to translate and validate the GOHAI Finnish version from the original English version. As the population ages rapidly, there is a growing prevalence of oral health issues among older adults, leading to a higher demand for oral health care services. Using tools like the Geriatric Oral Health Assessment Index (GOHAI) can effectively gather information on these oral health challenges among older individuals.

**Materials and Methods:**

Translation of the original GOHAI version was performed by a forward‐backward process and tested by a questionnaire in three cities in southern and Eastern Finland in 2020–2021. Reliability was assessed by measuring internal consistency and validity through convergent validity, discriminant validity, and known‐group validity.

**Results:**

A total of 209 participants aged 65 years or over (84 males and 125 females) completed the GOHAI questionnaire. The mean GOHAI‐ADD score (±SD) was 49.9 ± 6.2 (range 31–60). The Cronbach's alpha coefficient for the Finnish version of GOHAI was 0.79, indicating a high level of internal consistency. Item‐total score correlations were between 0.26 and 0.63. The use of removable dentures, perceived need for oral health care, and toothache or other problems with teeth or dentures were associated with poorer oral health‐related quality of life, whereas good self‐perceived oral health was associated with better oral health‐related quality of life.

**Conclusion:**

The Finnish version of the GOHAI showed good reliability and moderate validity among older people who had sought oral health care. However, the study design, including its cross‐sectional nature, reliance on a convenience sample, and the absence of clinical examinations, limits the generalizability of the findings to all older adults in Finland.

## 1 Introduction

The Finnish population is aging quickly and Finland is one of the global leaders in population ageing (Ageing Europe—Statistics on Population Developments 2026). At present, 23% of the population is aged 65 or above, and by the year 2050, more than every fourth Finn is predicted to be in this age group (Statistics Finland). This trajectory presents significant challenges to health and social care, necessitating comprehensive knowledge of various aspects of geriatric well‐being and older adults’ functional ability.

Oral health is a fundamental component of overall health, the quality of life and particularly important in the context of ageing. According to WHO, good oral health consists of good functionality and includes psychosocial dimensions such as self‐confidence, well‐being, and the ability to socialize and work without pain, discomfort, and embarrassment (World Health Organization 2022). The aim in assessing the oral health‐related quality of life (OHRQoL) is to complement clinical examinations with the individual's perception of oral‐related factors. Although there are different definitions of OHRQoL, the following four components are generally included: functioning, pain/discomfort, psychological aspects, and social aspects. OHRQoL instruments should illuminate all these and be suitable for growing older adult populations.

Azami‐Aghdash et al. (2021) systematic review and meta‐analysis highlight the inadequacy of oral health‐related quality of life (OHRQoL) measures for older adults. In Finnish population studies (Torppa‐Saarinen et al. 2021), the most frequently used OHRQoL instrument has been the Oral Health Impact Profile (OHIP). This widely used questionnaire provides good comparable data, but many studies indicate that the Geriatric Oral Health Impact indicator (GOHAI) developed by Atchison and Dolan (1990) would be a more suitable instrument to measure OHRQoL in older adult populations. However, a recent systematic review has evaluated the OHIP instrument slightly ahead of the GOHAI when using the Evaluating Measures of Patient‐Reported Outcomes tool (Deana et al. 2024). Globally, specific instruments that have undergone rigorous validation are limited, with GOHAI, OHIP‐14, and The Oral Impact on Daily Performance standing out as the most widely validated (Riva et al. 2022). The GOHAI, a compact self‐reported measure based on 12 questions, has undergone validation in several European countries, including Sweden (Hägglin et al. 2005), Germany (Hassel et al. 2008), France (Tubert‐Jeannin et al. 2003), Spain (Aguirre‐Bustamante et al. 2020), and the Netherlands (Niesten et al. 2016).

In Finland, the TOIMIA Functioning Measures Database offers an open‐access and free‐of‐charge tool for experts and professionals to evaluate functioning in clinical practice and research. However, the current database lacks assessment tools for evaluating the oral health status of older adults (Finnish Institute for Health and Welfare 2026). To enhance oral health for older adults in Finland, we need validated measurement tools tailored to their specific needs, comprehensively assessing oral functional capacity.

The aim of our study was to translate the original English version of GOHAI into Finnish and validate the translated indicator in Finland, and subsequently incorporate the GOHAI indicator into TOIMIA Functioning Measure database.

## 2 Materials and Methods

### 2.1 Translation

The validation process for the Finnish translation of the GOHAI form started in 2017. Permission was sought and obtained from Professor Atchinson to translate the form in November 2017. After having received the approval in May 2019, two individuals who were specialists in oral health produced their own independent Finnish translations of the instrument, which were then harmonized into a single version by the validation working group. The consolidated translation was back‐translated into English in June 2019 to assess the accuracy and equivalence of the translation. To evaluate the linguistic and conceptual consistency of the translated instrument, a native English speaker with professional fluency in Finnish and who was also the so‐called naive translator (neither aware nor informed of the concept) conducted a back‐translation of the Finnish version into English. This step ensured that the translated items accurately reflected the meaning and intent of the original instrument. A new Finnish version was subsequently derived by authors A.L.S. and K.S. based on the original and the two translated versions. The combined Finnish translation was translated back into English in June 2019. In September, the accuracy of the translation was verified by comparing the original and the English translation. The authors, A.L.S., K.S., and M.Ä., then decided on the final translated version for the pilot study.

### 2.2 Study Design

The data for the study were collected in southern and Eastern Finland. The target group consisted of people over 65 years of age who sought oral health care from September 2020 to May 2021 from three different localities: the City of Espoo (southern Finland) and the Cities of Kuopio and Iisalmi (Eastern Finland). A convenience sample was used to gather at least 70 participants from each of the three cities to get enough statistical power.

This study was planned and conducted following the instructions of the University of Eastern Finland (UEF) Committee for Research Ethics (Research ethics | University of Eastern Finland) (https://www.uef.fi/en/research-ethics), which evaluates the ethicality of non‐medical research projects. Informed written consent was collected from each participant. In addition, research permissions were obtained separately from each owner of the patient registers in all the three localities.

In the city of Espoo, the data were collected from patients over 65 years of age in the Haukilahti dental clinic. Due to the COVID‐19 epidemic, oral health care for Espoo residents over 70 years of age was centralized in two dental clinics until at least 1st June 2021, of which Haukilahti Dental Clinic was one. The office nurse determined the patient's willingness to participate in the study, gave the patient the study information sheet, the background information form, and the actual 12‐question GOHAI form. The patient filled in the forms in the waiting room, and the form was checked by an oral health care professional (dentist, oral hygienist, or dental nurse).

In the city of Kuopio, the data were collected from patients over 65 years of age in the Oral and Maxillofacial Teaching Clinic of the Pohjois‐Savo Hospital District. The oral hygienist/dental student trainee at the clinic ascertained the patient's willingness to participate in the study and recorded this in the patient's medical record. This was to ensure that the patient was not asked more than once to participate in the study. If the patient consented to the study, the student coded the consent form, the background information form and the GOHAI form and gave them to the patient to complete during the appointment. The student checked that the forms had been completed correctly or, if necessary, assisted the participant in completing the forms.

In the city of Iisalmi, the data were collected from oral health care patients over 65 years of age in the various clinics of the municipality during their appointments. The patient's willingness to participate in the study was determined by oral health professional. The patient's medical record was marked “GOHAI” to indicate that the patient has been offered the opportunity to participate in the study. The patient participating in the study filled in the study forms either at the clinic with the oral health professional or independently in the waiting room. If the patient wished, he/she could ask the office nurse or other oral health professional for help if he/she was unable to complete the forms independently. Informed written consent was collected from each participant.

### 2.3 Methods

The participants were first asked to provide background information. The background form included the following questions about oral and general health: 1. How do you perceive your oral health? (very poor, poor, moderate, good, very good); 2. Have you had toothache or other tooth or denture‐related problems in the past 12 months? (yes/no); 3. Do you think you need dental treatment at the moment? (yes/no); 4. How do you perceive your general health? (very poor, poor, moderate, good, very good); 5. Do you use removable dentures? (Yes, full prosthesis in the upper jaw, Yes, full prosthesis in the lower jaw, Yes, partial prosthesis in the upper jaw, Yes, partial prosthesis in the lower jaw, No full or partial prosthesis); 6. How many own teeth do you have? (less than 5, 5–10, 11–20, 21–32). Quality of life was assessed by two questions: How do you perceive your quality of life at the moment? (very poor, poor, moderate, good, very good); What affects your quality of life the most? (oral health problems, other health problems, low social contacts, financial situation). In addition, date of birth, gender, and place of residence were recorded. Socioeconomic status (SES) (high, middle, low) was assessed based on level of education. The total annual income of the household was asked and was divided into categories (less than 10,000€, 10,000€–19,999€, 20,000€–29,999€, 30,000€–39,999€, over 40,000€).

After having completed the background form, the GOHAI form was used to assess participants’ oral health‐related quality of life. The form included 12 questions (each question addressing one oral health item). Respondents were asked how often, in the previous 3 months, they had experienced the oral health item addressed with answering options “always,” “often,” “sometimes,” “seldom,” or “never.” Answers for the form were received from 214 patients. Subjects who had two or more missing GOHAI answers were excluded. In the case of one missing GOHAI answer (Atchison and Dolan 1990), the average of the person's responses was calculated, and the missing response was replaced by this one. The final number of the responses was 209 of which 24 had needed assistance in filling in the form.

## 3 Analyses

### 3.1 General Psychometric Properties

The percentage of responses to each GOHAI item, as well as to the GOHAI‐ADD (additive) score and the GOHAI‐SC (simple count) score (Atchison and Dolan 1990), was computed.

#### 3.1.1 The GOHAI‐ADD (Additive) Score

Each GOHAI‐item was scored from 1 to 5, with higher scores indicating better OHRQoL except for questions 3, 5, and 7. Therefore, scores for questions 3, 5, and 7 were reversed so that all items scored in the same direction. The GOHAI‐ADD score was then calculated by summing all item scores (total score ranging from 12 to 60), higher score indicating better oral health‐related life.

#### 3.1.2 The GOHAI‐SC (Simple Count) Score

The GOHAI‐SC score was calculated by summing all items answered as “always,” “often,” or “sometimes,” which were scored as 1. Conversely, responses of “seldom” or “never” were scored as 0. Each item was therefore scored either 0 or 1, a score of 1 indicating impairment for that item, resulting in a total score ranging from 0 to 12 higher score indicating poorer oral health‐related life.

Floor and ceiling effects were evaluated based on the total scores for both GOHAI‐ADD and GOHAI‐SC. These effects were deemed significant if 20% or more of the participants achieved either the lowest possible score (floor effect) or the highest possible score (ceiling effect) (Nunnally and Bernstein 1994).

### 3.2 Reliability

Reliability was evaluated by examining internal consistency, which involved assessing the correlation between the 12 individual GOHAI item scores and the total GOHAI‐ADD score. This was done using corrected item‐total score correlation (Spearman's rank correlation coefficient) and Cronbach's alpha. Item‐total score correlation is a statistical measure used in psychometrics and questionnaire validation. It indicates how well each individual item (question) on a test or survey correlates with the total score of the entire scale (excluding that item). In other words, it checks whether an item is consistent with the overall construct being measured. A high item‐total correlation (typically ≥ 0.3 or 0.4) means the item aligns well with the overall scale. A low correlation (typically < 0.2) suggests the item may not measure the same concept as the rest of the scale and might need revision or removal. Cronbach's alphas above 0.7 are generally seen as acceptable for group‐level comparisons, while alphas exceeding 0.9 are considered suitable for individual‐level comparisons, indicating high consistency (Nunnally and Bernstein 1994; Fayers and Machin 2000). Cronbach's alphas above 0.4 suggest adequate consistency between items within a subscale, while alphas surpassing 0.7 indicate adequate consistency between the subscale and the overall scale (total score) (Fayers and Machin 2000). Inter‐item correlations were calculated to assess how closely the items were associated with each other (ideally, the average inter‐item correlation falls between 0.2 and 0.5 (Fayers and Machin 2000; Clark and Watson 1995) and to identify the redundancy of items (Cronbach's alpha above 0.7) (Ponterotto and Ruckdeschel 2007). The dimensional structure of the GOHAI was evaluated through assessment of correlations between item scores and the GOHAI‐ADD score of the related dimension (subscale). Cronbach's alphas > 0.4 are considered indicative for adequate item – subscale consistency, and Cronbach's alphas > 0.7 are considered indicative for adequate subscale ‐ overall scale (total score) consistency (Nunnally and Bernstein 1994). Stability was not possible to assess by measuring test‐retest reliability since this study was based on one cross‐sectional sample.

### 3.3 Validity

Construct validity was evaluated through convergent validity, discriminant validity, and known‐group validity. Convergent validity examines the extent to which two measures, designed to measure the same construct, are correlated. This was evaluated by assessing the correlations between GOHAI‐ADD scores and the answers to a general question on self‐perceived oral health. A high correlation was anticipated between higher GOHAI‐ADD scores and better self‐perceived oral health. Discriminant validity assesses how two measures, aimed at similar but conceptually distinct constructs, correlate. This was evaluated by examining the correlation between the GOHAI‐ADD scores and perceptions of general health. A moderate correlation was anticipated between higher GOHAI‐ADD scores and better self‐perceived general health. Known‐group validity measures how well a test can detect differences between subgroups expected to vary in scores. This was evaluated by comparing GOHAI‐ADD scores across subgroups based on gender, age, education level, income, number of own teeth, use of removable dentures, treatment need, toothache or other problems with teeth or dentures, perceived oral health, perceived health, and perceived quality of life. Participants who did not perceive a need for treatment, had more own teeth, did not use removable dentures, did not report toothache or other problems with teeth or dentures, and perceived their oral and other health as good were expected to have higher GOHAI‐ADD scores. For comparisons, Spearman's rank correlation coefficients (*r*), where values above 0.5 indicate a strong correlation, 0.35 to 0.5 indicate a moderate correlation, and 0.2–0.34 indicate a low correlation (Bearden and Netemeyer 1999; Juniper and Gordon 1996), and Mann–Whitney *U*/Kruskal–Wallis (nonparametric) tests were used. In addition, associations of sociodemographic, socioeconomic, oral health‐related and health‐related factors with Geriatric Oral Health Assessment Index (GOHAI) additive (GOHAI‐ADD) and simple (GOHAI‐SC) scores were analyzed by multivariable linear regressions. In these analyses the GOHAI‐ADD and GOHAI‐SC served as outcomes, and sociodemographic, socioeconomic, oral health‐related, and health‐related factors as explanatory factors adjusted for each other. Positive *β* estimates indicate a positive association between the explanatory and outcome variables, whereas negative estimates indicate an inverse association.

Statistical analyses were conducted using IBM SPSS Statistics (version 27).

## 4 Results

Of the respondents, 59.8% were female, 57.1% had a minimum of 20 own teeth, and 30.3% had a removable denture. The level of education was reported being high in 24.9% of the participants, medium in 47.4%, and low in 27.8% (Table 1).

**Table 1:** Characteristics of the participants.

	*n*	%
All	209	100
**Gender**		
Male	84	40.2
Female	125	59.8
**Age group (years)**		
65–70	79	38.0
71–74	67	32.2
7–80	38	18.2
81 and over	24	11.6
Missing	1	
**Level of education**		
High	52	24.9
Middle	99	47.3
Low	58	27.8
**Household income (€)**		
< 10,000	14	6.8
10,000–19,999	38	18.4
20,000–29,999	38	18.4
30,000–39,999	54	26.2
40,000 and over	62	30.2
Missing	3	
**Place of residence**		
Espoo	67	32.1
Kuopio	65	31.1
Iisalmi	58	27.7
Other	19	9.1
**Number of own teeth**		
Less than 5	11	5.5
5–10	29	14.4
11–20	45	22.4
21–32	116	57.7
Missing	8	
**Removable denture**		
Yes	61	30.3
No	140	69.7
Missing	8	
**Perceived need for dental treatment**		
Yes	146	71.8
No	57	28.2
Missing	5	
**Perceived oral health**		
Very good	17	8.2
Good	98	47.1
Moderate	83	39.9
Poor	10	4.8
Very poor	—	—
Missing	1	
**Toothache or other problems with teeth or dentures**		
Yes	97	46.9
No	110	53.1
Missing	2	
**Perceived health**		
Very good	17	8.2
Good	111	53.9
Moderate	70	34.0
Poor	7	3.4
Very poor	1	0.5
Missing	3	
**Perceived quality of life**		
Very good	30	14.5
Good	118	57.0
Moderate	54	26.1
Poor	4	1.9
Very poor	1	0.5
Missing	2	

### 4.1 General Psychometric Properties

Answer proportions and the percentage of impairment (based on the number of answers “always,” “sometimes,” or “often”) are listed in Table 2. The mean total score (GOHAI‐ADD) was 49.9 ± 6.2 (range 31–60), and the mean simple count score (GOHAI‐SC) was 2.7 ± 2.4 (range 0–11). The items that showed the highest frequency of impairment were item 9 (54.5%), item 7 (41.4%), and item 5 (34.5%). The items that showed the lowest frequency of impairment were items 6, 4, and 11.

**Table 2:** Answer proportions (%) to each Geriatric Oral Health Assessment Index (GOHAI) item and proportion of those participants (*n* = 209) scoring “impairment.”^a^

GOHAI item	Never	Seldom	Sometimes	Often	Always	Impairment^a^
	**%**					
1. Limit the kinds of food	52.0	29.7	11.4	6.4	0.5	18.3
2. Trouble biting or chewing	42.4	31.5	16.7	7.9	1.5	26.1
3. Able to swallow comfortably	5.5	6.5	2.8	14.9	70.6	14.8
4. Unable to speak clearly	81.3	13.8	3.0	2.0	0.0	5.0
5. Able to eat without discomfort	11.8	16.3	6.4	12.3	53.2	34.5
6. Limit contact with people	86.7	10.3	2.0	1.0	0.0	3.0
7. Pleased with look of teeth	3.9	14.3	23.2	34.5	24.1	41.4
8. Used medication to relieve pain	45.3	41.9	11.8	1.0	0.0	12.8
9. Worried about teeth, gums or dentures	12.9	32.7	39.6	13.4	1.5	54.5
10. Self‐conscious of teeth, gums or dentures	43.3	34.0	17.2	3.0	2.5	22.7
11. Uncomfortable eating in front of others	66.5	24.1	5.4	3.4	0.5	9.3
12. Sensitive to hot, cold or sweet foods	19.7	47.8	27.1	5.4	0.0	32.5

*Note*: Participants with two or more GOHAI answers missing were excluded. In case only one GOHAI answer was missing, the missing value was replaced by mean substitution.

^a^ Combined answers “always,” “sometimes,” and “often”; reverse coded for items 3, 5, and 7.

No floor or ceiling effects were detected at the GOHAI‐ADD: 1.4% had the highest possible score of 60, and none had the lowest possible score of 12. The GOHAI‐SC score, however, did show a floor effect: 20.6% had a total score of zero.

### 4.2 Reliability

Cronbach's alpha was 0.79, indicating good overall internal consistency. The corrected item‐total score correlations were between 0.26 and 0.63, as shown in Table 3. All other items except 5, 8, and 12 showed high (≥ 0.30 or 0.40) correlations. However, also correlations for items 5, 7, and 12 were above low. For only one item (Item 5), the Cronbach's alpha if the item was deleted was slightly higher than the overall alpha of 0.79.

**Table 3:** Reliability analysis between the 12 individual Geriatric Oral Health Assessment Index (GOHAI) item scores and the total GOHAI‐ADD^a^ score: Corrected^b^ item‐total score correlation and Cronbach's alpha.

GOHAI item	Corrected^b^ item‐total score correlation	Cronbach's alpha if item deleted
1. Limit the kinds of food	0.61	0.75
2. Trouble biting or chewing	0.60	0.75
3. Able to swallow comfortably	0.34	0.78
4. Unable to speak clearly	0.51	0.77
5. Able to eat without discomfort	0.29	0.80
6. Limit contact with people	0.46	0.77
7. Pleased with look of teeth	0.33	0.78
8. Used medication to relieve pain	0.26	0.78
9. Worried about teeth, gums or dentures	0.55	0.76
10. Self‐conscious of teeth, gums or dentures	0.63	0.75
11. Uncomfortable eating in front of others	0.59	0.76
12. Sensitive to hot, cold or sweet foods	0.28	0.78

^a^ Additive GOHAI‐ADD score is the sum of all scores (score 1–5 per answer; total score ranging from 12 to 60), a higher score indicates better oral health‐related life.

^b^ Excluding the item in question.

Inter‐item correlations were generally within the acceptable range of 0.2–0.5; however, higher correlations were observed between items 1 and 2 (*r* = 0.73), items 9 and 10 (*r* = 0.67), and items 10 and 11 (*r* = 0.51).

The dimensional structure of the original GOHAI was only partly supported by Cronbach's alphas and item‐subscale correlation values (Table 4). Cronbach's alphas for subscale—overall scale correlations were above the threshold of 0.7 for the dimensions “physical functioning” and “psychosocial functioning,” and all item—subscale correlations within these dimensions were adequate (above > 0.45) except for items 6 and 7. The dimension “pain and discomfort” showed inadequate (< 0.7) subscale, overall scale consistency, and all items (3,5,8,12) within this dimension were only weakly correlated to the dimension total score (Cronbach's alphas between 0.16 and 0.42).

**Table 4:** Correlation between item‐subscale (dimension) scores and between subscale‐overall scale scores.

GOHAI items and dimensions	Cronbach's alpha
**Dimension: Physical functioning subscale‐overall scale**	0.80
1. Limit the kinds of food	0.78
2. Trouble biting or chewing	0.77
4. Unable to speak clearly	0.47
**Dimension: Pain and discomfort subscale‐overall scale**	0.45
3. Able to swallow comfortably	0.42
5. Able to eat without discomfort	0.32
8. Used medication to relieve pain	0.17
12. Sensitive to hot, cold or sweet foods	0.16
**Dimension: Psychosocial functioning subscale‐overall scale**	0.74
6. Limit contact with people	0.42
7. Pleased with look of teeth	0.35
9. Worried about teeth, gums or dentures	0.58
10. Self‐conscious of teeth, gums or dentures	0.69
11. Uncomfortable eating in front of others	0.56

### 4.3 Validity

Table 5 shows the main results of comparisons between assumedly construct‐related variables and GOHAI‐ADD scores. Moderate correlation (0.41) in the expected directions was found between the GOHAI‐ADD score and self‐perceived oral health. However, those reporting good or very good perceived oral health reported higher scores than those reporting moderate or poor oral health. Lower (0.29) correlation in the expected direction was found between the GOHAI‐ADD score and self‐perceived general health. The only low correlations observed were between the gender, age or level of education and GOHAI‐ADD scores. In addition, participants who reported a higher number of teeth and good quality of life and had neither removable dentures nor a need for dental treatment, toothache, or other denture‐related problems scored statistically significantly higher than those who reported fewer teeth, poorer quality of life, had dentures, required dental treatment, or experienced toothache or other denture‐related problems.

**Table 5:** Validity assessments: Spearman's rank correlations between selected variables and Geriatric Oral Health Assessment Index additive (GOHAI‐ADD)^a^ scores.

Variable	*n*	Mean GOHAI‐ADD^a^ score (SD)	Correlation coefficient^b^	*p*‐value
**Gender**				
Male	84	50.07 (6.35)	0.04	0.55^c^
Female	125	49.74 (6.19)		
**Age group (years)**				
65–70	79	49.47 (6.38)	0.02	0.27^d^
71–74	67	50.25 (5.75)		
75–80	38	51.18 (6.49)		
81 and over	24	48.29 (6.59)		
**Level of education**				
Low	58	49.76 (6.87)	0.09	0.04^d^
Middle	99	49.09 (5.84)		
High	52	51.48 (6.05)		
**Household income (€)**				
< 20,000	52	48.77 (7.21)	0.16	0.08^d^
20,000–29,999	38	48.61 (7.05)		
30,000–39,999	54	49.83 (5.51)		
40,000 and over	62	51.65 (5.06)		
**Number of own teeth**				
Less than 5	11	49.19 (5.93)	0.24	0.01^d^
5–10	29	46.48 (7.74)		
11–20	45	48.49 (6.44)		
21–32	116	51.37 (5.39)		
**Removable denture**				
Full denture upper jaw	32	48.31 (7.09)	0.25	< 0.001^d^
Full denture lower jaw	—	—		
Partial denture upper jaw	25	45.36 (6.97)		
Partial denture lower jaw	4	46.75 (5.68)		
No removable denture	140	51.05 (5.56)		
**Perceived need for dental treatment**				
Yes	146	48.56 (6.44)	0.30	< 0.001^c^
No	57	52.82 (4.64)		
**Perceived oral health**				
Very poor	0	—	0.41	< 0.001^d^
Poor	10	40.50 (6.45)		
Moderate	83	48.05 (6.00)		
Good	98	51.69 (5.28)		
Very good	17	53.71 (4.59)		
**Toothache or other problems with teeth or dentures**				
Yes	97	47.45 (6.09)	0.38	< 0.001^c^
No	110	51.95 (5.64)	0.38	< 0.001^c^
**Perceived health**				
Very poor	1	50.00 (−)	0.29	0.001^d^
Poor	7	44.57 (8.26)		
Moderate	70	48.17 (6.44)		
Good	111	50.76 (5.87)		
Very good	17	53.53 (4.19)		
**Perceived quality of life**				
Very poor	1	50.00 (−)	0.32	< 0.001^d^
Poor	4	42.25 (9.43)		
Moderate	54	47.46 (6.46)		
Good	118	50.20 (5.96)		
Very good	30	53.67 (4.51)		

Abbreviation: SD, standard deviation.

^a^ GOHAI‐ADD score is the sum of all scores (score 1–5 per answer; total score ranging from 12 to 60), a higher score indicates better oral health‐related life.

^b^ Spearman's rank correlation coefficient.

^c^ From the Mann–Whitney *U* test.

^d^ From the Kruskal–Wallis test.

Based on regression analyses (Table 6), perceived good oral health is associated with better OHRQoL, that is positively with a higher GOHAI‐ADD score and inversely with a higher GOHAI‐SC score. Having removable dentures, perceived need for oral health care, and toothache or other problems with teeth or dentures were associated with poorer oral health‐related life, whereas good self‐perceived oral health was associated with better OHRQoL.

**Table 6:** Associations of sociodemographic, socioeconomic, oral health‐related and health‐related factors with Geriatric Oral Health Assessment Index (GOHAI) additive (GOHAI‐ADD^a^) and simple (GOHAI‐SC^b^) scores analyzed by multivariable linear regressions.

	GOHAI‐ADD^a^			GOHAI‐SC^b^		
	Β (95% CI)	SD	*p*‐value	Β (95% CI)	SD	*p*‐value
Female gender (ref. males)	−0.22 (−1.90 to −1.45)	0.85	0.794	−0.11 (−0.74 to −0.11)	0.32	0.729
Age group (years)^c^	0.69 (−0.14 to −1.53)	0.42	0.104	−0.20 (−0.52 to −0.40)	0.16	0.211
Educational level^d^	0.20 (−1.02 to −1.43)	0.62	0.743	−0.06 (−0.52 to −0.40)	0.23	0.799
Household income^e^	0.24 (−0.58 to −1.05)	0.41	0.566	−0.15 (−0.45 to −0.16)	0.16	0.338
Place of residence^f^	0.19 (−0.72 to −1.09)	0.46	0.685	0.09 (−0.25 to −0.43)	0.17	0.608
Number of own teeth^g^	0.57 (−0.60 to −1.74)	0.59	0.336	−0.17 (−0.61 to −0.27)	0.22	0.452
Removable dentures (ref. no)	**−2.28 (−4.67** to **−0.11)**	**1.21**	**0.061**	0.71 (−0.19 to −1.60)	0.46	0.123
Perceived need for oral health care (ref. no)	**−2.64 (−4.56** to **−0.72)**	**0.97**	**0.007**	**1.19 (0.47–1.91)**	**0.37**	**0.001**
Perceived good oral health (ref. moderate or poor)	**2.78 (0.98–4.43)**	**0.88**	**0.002**	**−1.28 (−1.92** to **−0.62)**	**0.33**	**< 0.001**
Toothache or other problems with teeth or dentures (ref. no)	**−2.70 (−4.39** to **−1.00)**	**0.86**	**0.002**	**0.77 (0.13–1.41)**	**0.32**	**0.018**
Perceived good health (ref. moderate or poor)	0.30 (−1.61 to −2.20)	0.96	0.758	−0.30 (−1.02 to −0.42)	0.36	0.409
Good quality of life (ref. moderate or poor)	1.23 (−0.81 to −3.27)	1.03	0.235	−0.45 (−1.22 to −0.32)	0.39	0.250

*Note*: β (95%CI) = Unstandardized β (95% confidence interval), SD=standard deviation. *p*‐value based on *t*‐test. Bold values indicate statistical significance.

^a^ GOHAI‐ADD score is the sum of all scores (score 1–5 per answer; total score ranging from 12 to 60), a higher score indicates better oral health‐related life.

^b^ GOHAI‐SC score is the sum of all items with response “always,” “sometimes,” and “often” as score 1 and “seldom” or “never” as score 0 per answer; total score from 0 to 12, where a ‘1’‐ score indicates impairment for that item and a higher sum score indicates poorer oral health‐related life.

^c^ Age group (years): 1 = 65–74, 2 = 71–74, 3 = 75–80, and 4 = 81+.

^d^ Educational level: 1 = elementary school, 2 = high school, and 3 = vocational school.

^e^ Household income: 1 = < 20,000 €, 2 = 20,000–29,999 €, 3 = 30,000–39,999 €, and 4 = ≥ 40,000 €.

^f^ Place of residence: 1 = Espoo, 2 = Kuopio, 3 = Iisalmi, and 4 = other.

^g^ Number of own teeth: 1 = < 5, 2 = 5–10, 3 = 11–120, and 4 = 21–32.

## 5 Discussion

### 5.1 Principal Findings

This study shows that GOHAI has good reliability and moderate validity and can be used to measure OHRQoL in the target group of the present study. However, aspects of the study's design—including its cross‐sectional nature, reliance on a convenience sample, and lack of clinical examinations—limit the generalizability of the findings in all Finnish older adults.

### 5.2 Strengths and Weaknesses of the Study

Regarding the strengths, a satisfactory number of responses to the questionnaire were received, which enabled the analyses needed. Furthermore, there was more variation in our sample than, for example, in the original study (Atchison and Dolan 1990). Three cities included in this study represented geographically different parts of Finland implicating also differences in oral health and socioeconomic situation. The localities included a large city in southern Finland, a medium size, and a small rural town in Eastern Finland. Since there were no exclusion criteria, participants represented all socio‐economic and health groups. However, due to the design of the study, a cross‐sectional convenience sample, results cannot be generalized to all older adults in Finland.

As another limitation of this study, the test–retest reliability (Atchison and Dolan 1990) was not measured, since the data were collected cross‐sectionally at only one time point. The practical implication is that the instrument's temporal stability remains unconfirmed. Moreover, all measurements properties (e.g., reliability, validity) were assessed only through participants’ subjective reports, without comparison to clinical examinations. The instrument's accuracy in reflecting clinical or functional status remains unconfirmed, and the instrument could be more suitable for research and population‐level assessment than for standalone clinical evaluation. However, for subjective constructs (e.g., quality of life, pain, perceived health), self‐reported data are often the most appropriate source.

Additionally, the study included only participants who had actively sought treatment, and therefore did not capture the perspectives of individuals who had not. This may mean that current validation does not apply for populations with limited access to care or higher levels of frailty. The sample may not capture their full range of functional, cognitive, socioeconomic, or health‐related challenges, such as or barriers to accessing care or the impact of multimorbidity, reduced mobility, and cognitive impairment. Reliability or sensitivity may be overestimated since they are validated on a group that is generally more health‐literate, help‐seeking, and capable of participating in assessments. We did not collect any information on patients who declined to participate, nor on their reasons for refusing participation in the study.

The corrected item–total correlation indicates how well an individual item correlates with the sum of the remaining items in the same scale. In our study, corrected item–total correlations for items 5 (able to eat without discomfort), 8 (used medication to relieve pain), and 12 (sensitive to hot, cold or sweet foods) were below 0.30, suggesting that these items do not align as good as the other items with the underlying construct measured by the scale. They probably refer to something else that is intended to be measured by the GOHAI, which is OHRQoL. There may be several reasons for this during the validation process: the items were poorly worded or ambiguous, the items were reverse‐coded incorrectly, or respondents have interpreted the items differently (e.g., cultural or language issues). However, all the values were above 0.20 and therefore considered as a warning sign, but not an automatic reason to discard an item. Moreover, the average inter‐item correlations were within the acceptable range (0.2–0.5) (Sánchez‐García et al. 2010; Atieh 2008), and the Cronbach's alpha exceeded 0.7, indicating no redundancy among items (Fayers and Machin 2000; Clark and Watson 1995; Ponterotto and Ruckdeschel 2007).

### 5.3 Strengths and Weaknesses in Relation to Other Studies

As far as results are concerned and compared to other similar studies, the mean GOHAI‐ADD score of 49.9 ± 6.2 in this study was at the same level with those found in Northwestern Europe and the United States (53 in Germany, 49.8 in Sweden, 46.4 in France, and 52.5 in the United States) (Hägglin et al. 2005; Hassel et al. 2008; Tubert‐Jeannin et al. 2003; Hassel et al. 2011), but for the most part higher than elsewhere (mean GOHAI‐ADD scores between 18 and 49 in Romania, Hongkong, Japan, Malaysian, Jordan, Turkey, India, Spain, Mexico, Iran, Persia, Nepal, and Saudi Arabia). Reliability was also found to be on a good level. Internal consistency (Cronbach's alphas of 0.79) was good and comparable with values of other (Hägglin et al. 2005; Hassel et al. 2008; Tubert‐Jeannin et al. 2003; Rodakowska et al. 2014; Rezaei et al. 2016; Sánchez‐García et al. 2010; Atieh 2008; Ergül and Akar 2008; Naito et al. 2006) GOHAI studies. Also, item‐total correlations in our study (0.26–0.61) were at the same levels as in Germany, Spain, Netherlands, Turkey, and Brazil (Hassel et al. 2008; Aguirre‐Bustamante et al. 2020; Niesten et al. 2016; Ergül and Akar 2008; Vettore et al. 2022) but lower than in Sweden (Hägglin et al. 2005) or in France (Tubert‐Jeannin et al. 2003).

Unlike OHIP (Rodakowska et al. 2014; Locker et al. 2001; Ikebe et al. 2012), the GOHAI questionnaire did not show floor and ceiling effects when looking at the GOHAI‐ADD scores, which are most frequently used for comparing groups in studies that utilize the GOHAI questionnaire. The GOHAI‐SC score, however, did show a floor effect: 20.6% had a total score of zero. The percentage is slightly lower than in the Dutch or French version (Tubert‐Jeannin et al. 2003; Niesten et al. 2016), for example, but shows that the subscales aren't covering the full scale of people who might respond. This could make it more difficult to notice any changes in oral health over time, in other words, be less useful for longitudinal or intervention studies. Although the instrument is functioning as intended, the floor effect in the GOHAI‐SC indicates that the instrument has limited ability to discriminate among individuals with poor oral health–related quality of life and reduced sensitivity to detect change, particularly in severely affected populations.

The subscale (dimension)–overall scale as well as item‐subscale (dimension) correlations for the dimension ‘pain and discomfort’ were low in our study. A similar finding was reported in a Dutch (Niesten et al. 2016) and Brazilian (Vettore et al. 2022) validation studies, suggesting that it may be worthwhile to reconsider these dimensions or to adopt a one‐dimensional scale.

Regarding convergent validity, discriminant validity, and known‐group validity, our results were in line with those observed in Sweden (Riva et al. 2022), Germany (Hassel et al. 2008), France (Tubert‐Jeannin et al. 2003), Brazil (Vettore et al. 2022), Turkey (Ergül and Akar 2008), and Saudi Arabia (Atieh 2008). In studies from the Netherlands (Aguirre‐Bustamante et al. 2020) and Spain (Tubert‐Jeannin et al. 2003), the correlations between GOHAI‐ADD and oral and general health, as well as socioeconomic variables, were in the expected directions but somewhat higher.

### 5.4 Meaning of the Study: Possible Mechanisms and Implications for Clinicians or Policymakers

For older people, oral health as part of functional capacity is an essential aspect of quality of life. For example, people with memory problems forget to take care of their oral health, and people with mobility problems may not be able to take care take their oral health or seek care. Both constraints affect both oral self‐care and the use of oral health services (Christensen et al. 2017).

The aim of this study was to validate the GOHAI and confirm its suitability as an indicator of OHRQoL in Finnish older adults. In older adults, GOHAI offers several advantages over other OHQoL measures, such as the Oral Health Impact Profile (OHIP‐14). GOHAI was specifically designed for the geriatric population and focuses on functional and pain‐related aspects—issues highly relevant for ageing Finns. This functional focus is often more actionable in clinical decision‐making than OHIP's broader psychosocial framework. GOHAI's brief, straightforward structure makes it easy to administer in routine clinical practice. GOHAI includes only 12 items using a straightforward 5‐point Likert scale, making it quicker to administer in busy clinical settings and easier for older adults to understand—beneficial in primary care and public health screenings. The GOHAI measure developed is suitable for use by the healthcare sector in the TOIMIA database and is the first oral health indicator in that database. The now validated GOHAI can be used as a supplementary data source to clinical data, for example, for the diagnosis of oral diseases and oral health examinations. The use of the indicator will support both the informal caregivers and oral health professionals, but also geriatricians and nurses in assessing the need for oral health care of older clients (Atchison and Dolan 1990; Denis et al. 2017).

Evaluating OHRoL holds significance in public health as it enables the depiction of how oral health affects functioning and psychosocial well‐being in older citizens. This information is crucial for efficiently distributing healthcare resources and assessing the effectiveness of various healthcare or dental care initiatives.

## 6 Conclusion

Our study demonstrated that the GOHAI exhibits good reliability and moderate validity, making it a well‐suited to serve as the first oral health indicator in the TOIMIA database, offering benefits for all healthcare professionals working with older adults. However, further research is needed to examine the reliability of GOHAI in longitudinal settings or across multiple time points in order to confirm its stability. In addition, larger and more representative datasets that also include clinical examinations are required to evaluate their usefulness as an outcome assessment tool for oral health care in the Finnish older population.

## Author Contributions

Critical contributions to the study design, project coordination, and data acquisition: Annamari Nihtilä, Marja Äijö, Kaarina Sirviö, Tiina Lampi, and Anna Liisa Suominen. Analysis of the data: Olli Koskela and Anna Liisa Suominen. Drafting of the manuscript, interpretation of results, and revision of the manuscript: Olli Koskela, Annamari Nihtilä, Marja Äijö, Kaarina Sirviö, and Anna Liisa Suominen. The contributions of each author have made to the manuscript are in accordance with the ICMJE authorship guidelines.

## Funding

The authors received no specific funding for this work.

## Disclosure

Artificial Intelligence was employed to edit the text in discussion.

## Ethics Statement

This study was planned and conducted following the instructions of the University of Eastern Finland (UEF) Committee for Research Ethics (Research ethics | University of Eastern Finland) (https://www.uef.fi/en/research-ethics), which evaluates the ethicality of non‐medical research projects. Informed written consent was collected from each participant. In addition, research permissions were obtained separately from each owner of the patient registers in all the three localities.

## Conflicts of Interest

The authors declare no conflicts of interest.

## Data Availability

The data that support the findings of this study are available from the corresponding author upon reasonable request.
